# Fructose Consumption in Pregnancy and Associations with Maternal and Offspring Hepatic and Whole-Body Adiposity in Rodents: A Scoping Review

**DOI:** 10.1016/j.cdnut.2024.104510

**Published:** 2024-11-27

**Authors:** Grace Zhao, Sarah Chondon, Clint Gray, Sheridan Gentili, Meagan Stanley, Timothy RH Regnault

**Affiliations:** 1 Department of Obstetrics and Gynaecology, Western University, London, ON, Canada; 2 Department of Obstetrics and Gynaecology, Schulich School of Medicine, Western University, London, ON, Canada; 3 Gillies McIndoe Research Institute, New Zealand; 4 Department of Paediatrics & Child Health, University of Otago, New Zealand; 5 Teaching Innovation Unit, University of South Australia, Australia; 6 Western Libraries, Western University, London, ON, Canada; 7 Department of Physiology and Pharmacology, Western University, London, ON, Canada; 8 Division of Maternal, Fetal, and Newborn Health, Children’s Health Research Institute, London, ON, Canada

**Keywords:** fructose, preconception, pregnancy, offspring, hepatic adiposity, whole-body adiposity, rodents, scoping review

## Abstract

**Background:**

Excess fructose consumption has been linked to adverse metabolic health, including impaired hepatic function and increased adiposity. The early life period, including preconception, pregnancy, and the newborn period, are critical periods in determining later metabolic health. However, the impact of excess fructose intake during this time on maternal, fetal, and offspring hepatic and whole-body adiposity, is not well defined.

**Objectives:**

To understand the effects of maternal fructose consumption pre- and during pregnancy on maternal, fetal, and offspring hepatic and whole-body adiposity.

**Methods:**

A systematic search of MEDLINE, EMBASE, and Cochrane Central Register of Controlled Trials was performed up to October 4, 2024, to identify animal and human studies that focused on maternal fructose consumption pre- and during pregnancy on hepatic and whole-body adiposity in the mother, fetus, and offspring. Citations, abstracts, and full texts were screened in duplicate. Hepatic adiposity was defined as elevated hepatic triglycerides or overall hepatic lipid accumulation. Whole-body adiposity was defined as increased adipose tissue, serum lipids, or adipocyte hypertrophy.

**Results:**

After screening 2538 citations, 37 experimental rodent studies reporting maternal fructose consumption pre- and during pregnancy in rodents were included. No human studies met the inclusion criteria. Prenatal fructose exposure was associated with maternal (9 of 12) and offspring (7 of 11) whole-body adiposity. A high proportion of studies (13 of 14) supported the association between fructose during pregnancy and increased maternal hepatic adiposity. Fetal hepatic adiposity and elevated expression of hepatic lipogenic proteins were noted in 4 studies. Offspring hepatic adiposity was supported in 16 of the 20 articles that discussed hepatic results, with 5 studies demonstrating more severe effects in female offspring.

**Conclusions:**

Fructose consumption during pregnancy in rodent models is associated with maternal, fetal, and offspring hepatic and whole-body adiposity with underlying sex-specific effects. No human studies met the inclusion criteria.

**Registration number:**

H8F26 on Open Science Framework (https://doi.org/10.17605/OSF.IO/H8F26)

## Introduction

Fructose has become an increasingly major component of the carbohydrate content in diets due to its high sweetness value [[1], [2], [3]]. In the past 40 y, the ubiquitous presence of fructose in high-fructose corn syrup in the United States food supply has exposed consumers to higher fructose-to-glucose ratios than are considered safe [4]. Excessive fructose consumption is associated with nonalcoholic fatty liver disease (NAFLD) as well as obesity, hyperuricemia, and insulin resistance [[5], [6], [7]], which are key components of metabolic syndrome [[8], [9], [10], [11]]. A healthy diet during pregnancy is essential to support the optimal growth and development of the fetus. However, data show that fewer pregnant women are meeting current diet recommendations [12]. Studies highlight that these dietary changes are associated with the increased consumption of low–nutrient-dense foods, including fructose-sweetened foods, which now appear to be the major contributors to the total energy intake in women during pregnancy [[13], [14], [15]].

Metabolically, intestinal fructose uptake is mediated by the glucose transporter GLUT5 (Slc2a5) in the apical membrane and GLUT2 (Slc2a2) in the basolateral membrane [16]. Within the intestinal epithelial enterocytes, fructose is phosphorylated by ketohexokinase (KHK) and converted to glucose, lactate, glycerate, and other organic acids, in addition to modulating the composition of gut microbiota and transcription of GLUT5 for further uptake [17,18]. However, the majority of dietary fructose exits the enterocyte via GLUT2 to enter the portal vein and into the liver [18]. Here, fructose is extracted by hepatocytes, likely through the GLUT2 transporter, and phosphorylated by KHK to fructose-1-phosphate, and then its metabolites act as signaling molecules to activate metabolic transcriptional programs associated with glucose production, lipogenesis, and glycogen synthesis [16]. Importantly, hepatic fructose metabolism involves the activation of transcription factors including carbohydrate response element–binding protein (ChREBP) and sterol regulatory element–binding protein 1 (SREBP1c), both of which regulate enzymes involved in fructolysis, glycolysis, glucose production, lipogenesis, and very low–density lipoprotein packaging and export [19,20], processes that underly hepatic de novo lipogenesis [21]. This possibly leads to the development of NAFLD, which is a public health issue [22].

In pregnancy, fructose is transported via the hexose transporters GLUT8 (Scl2a8) and GLUT9 (Scl2a9), which are expressed in human placentae [23,24]. Fructose is an important placental product from glucose [25, [26], [27]], and also a substrate for normal placental development, playing a key role during the relatively hypoxic early placentation period to maintain ATP concentrations and cellular redox potentials [27,28]. In early gestation, fructose is preferred over glucose in relatively hypoxic tissues. It can be endogenously produced from glucose in some pathological conditions. Furthermore, fructose production normally decreases significantly following the establishment of maternal–fetal circulation following placentation [28]. The negative impacts of excessive fructose consumption have been demonstrated in nonpregnant human and animal studies [29,30]. In pregnant rodent models, excessive fructose consumption is associated with placental insufficiency, elevated uric acid synthesis, and abnormal fetal growth [31]. In humans, maternal serum fructose concentrations are correlated with a risk of gestational diabetes [[32], [33], [34]], pre-eclampsia [35], and elevated placental uric acid concentrations. This suggests that similar uric acid pathways exhibited in rodent models might also be functioning in human placenta [36]. Additionally, studies show that exposure to certain flavors through the amniotic fluid and breast milk has an influence on the development of taste preferences [37,38] and links with increased whole-body adiposity and NAFLD are emerging [39,40]. Although human studies have highlighted the adverse impact of high-fructose diets on metabolic health in the nonpregnant state, the understanding of fructose’s impact in the periconceptional and pregnant state, specifically on human maternal and offspring body adiposity and hepatic fat content as a major readout of metabolic health and association with metabolic syndrome, is still developing.

Animal and human studies have shown that excessive fructose intake during pregnancy has potential adverse effects on fetal development and pregnancy outcomes [41,42]. However, the short- and long-term impacts of elevated fructose consumption on hepatic and whole-body adiposity, early markers of later life metabolic health, in the early life period still needed to be elucidated. Therefore, we conducted a scoping review of the current literature to examine the relationship between fructose consumption during prepregnancy, pregnancy, and lactation, and its effects on maternal and offspring hepatic and whole-body adiposity in both human and animal studies.

## Methods

A scoping review was conducted with guidance from the scoping review methodological framework developed by Arksey and O’Malley [43], the Synthesis Without Meta-Analysis guideline, [44] and the JBI methodology for scoping review [45]. The protocol was prospectively registered with Open Science Framework (identifier H8F26).

### Search strategy

The search strategy was developed in consultation with a health sciences information specialist (MS) and searched MEDLINE (Ovid), EMBASE (Ovid), and Cochrane Central Register of Controlled Trials from inception to October 4, 2024, for human and animal studies with no language restrictions. The full search strategy can be found in Appendix A. Bibliographies of included studies and relevant reviews were reviewed manually to identify additional studies.

### Eligibility criteria

Study eligibility was defined using the following population, exposure, comparison group, outcome (PECO) criteria: *1)* pregnant dams or women; *2)* maternal fructose consumption (prepregnancy, during pregnancy to throughout lactation period); *3)* included a comparison group; and *4)* reported hepatic adiposity (liver weight, hepatic triglycerides, histological hepatic lipid droplets) or whole-body adiposity (adipose tissue, weight gain, serum lipids). Studies focusing on obstetrical outcomes, infant formula, abstracts, conference proceedings, letters to the editor, case reports, and literature reviews were excluded.

### Study selection

All identified citations were uploaded into Covidence, and duplicates were removed. Following a pilot test, abstracts, and full texts were assessed for eligibility in duplicate by independent reviewers (2 of GZ, SC, TRHR, CG, SG). Disagreements on study eligibility were discussed with the study team and resolved by consensus. Notably, the eligibility criteria were modified at the full-text screen such that studies that used fructose in combination with other diets instead of in isolation were excluded. The study selection process is depicted using the “Preferred Reporting Items for Systematic Review and Meta-Analyses” (PRISMA) diagram [96].

### Data extraction

Data extraction was performed by GZ and SC and verified by TRHR using a data extraction tool developed by the reviewers. We conducted a pilot extraction of 5 articles to address any concerns with the article selection criteria and the extraction tool. For included articles, we extracted study characteristics (*i.e*., year, region), population characteristics (i.e., animal species, follow-up timeline), exposure characteristics (*i.e*., fructose concentration and timing of administration), and hepatic fat accumulation or whole-body adiposity characteristics (maternal, fetal, placental, or offspring), and potential mechanisms of adiposity. Fructose exposure was categorized as high (>30% wt:vol or wt:wt) or low (<30% wt:vol or wt:wt) to simplify the variation of fructose concentrations. The threshold of 30% was chosen as studies have shown that concentrations above this exceed the average human intake and are not physiological [[46], [47], [48]]. We categorized hepatic adiposity as elevated hepatic triglyceride content and macro- and microvesicular fatty infiltration in hepatocytes. We considered whole-body adiposity as an increase in adipose tissue (retroperitoneal, visceral, white) or adipocyte hypertrophy. Data for hepatic and whole-body adiposity measures are presented in a narrative format with tables and figures.

## Results

### Study characteristics

**FIGURE 1:**
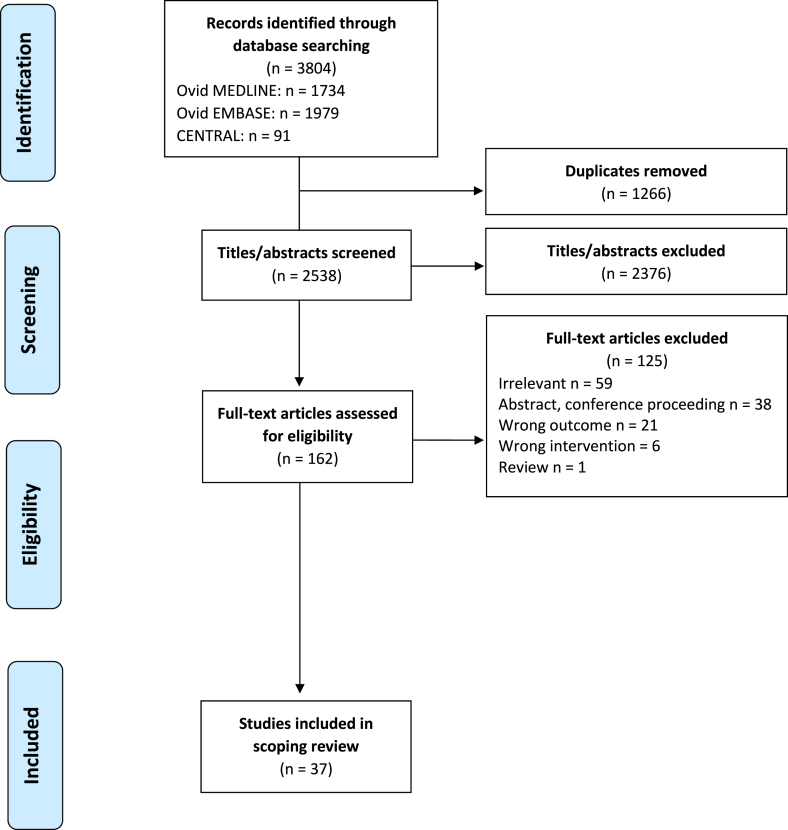
PRISMA flow diagram of literature search and selection process.

**FIGURE 2:**
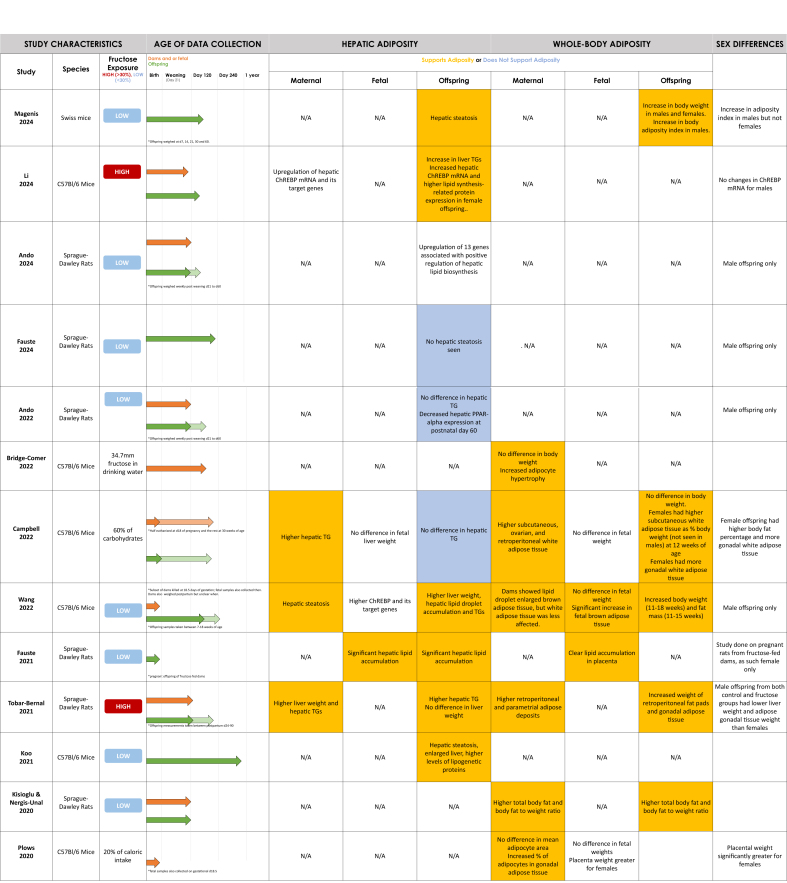

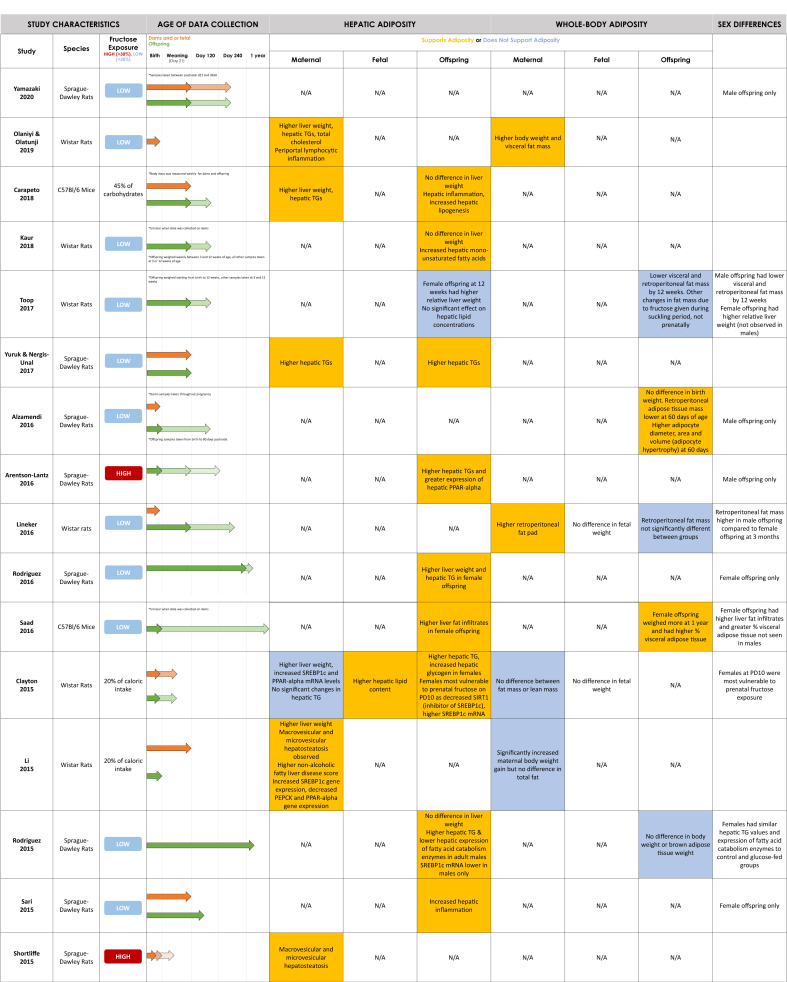

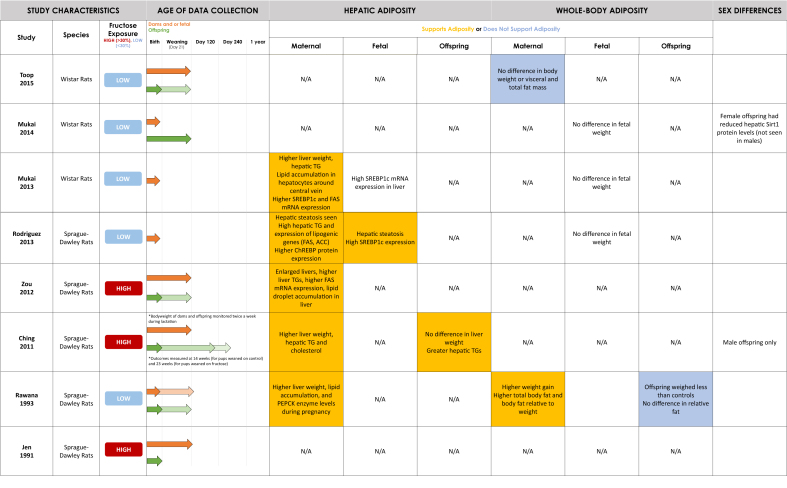
Summary of included study characteristics and hepatic and whole-body adiposity findings.

The systematic search yielded 2538 citations after duplicates were removed, of which 37 met the inclusion criteria (Figure 1). The included 37 records were published between 1990 and 2024, with 21 (57%) published between 2000 and 2019 (Table 1). Individual study characteristics are presented in Table 2 [39,40,[42], [43], [44], [45], [46], [47], [48], [49], [50], [51], [52], [53], [54], [55], [56], [57], [58], [59], [60], [61], [62], [63], [64], [65], [66], [67], [68], [69], [70], [71], [72], [73], [74], [75], [76], [77], [78], [79], [80], [81], [82]]. All included studies were experimental studies on rodents and no human reports met the inclusion criteria. Most included studies were conducted in North America (*n* = 8, 22%) and Oceania (*n* = 8, 22%). Although the timing and duration of fructose exposure differed between studies, the 2 most common timings were during pregnancy only (*n* = 13, 35%) and during pregnancy and lactation (*n* = 12, 32%). High-dose fructose solution (>30% wt:vol or wt:wt) was used in 7 studies [42,[49], [50], [51], [52], [53], [54]] and low-dose fructose solution (<30% wt:vol or wt:wt) was used in 24 studies [39,[55], [56], [57], [58], [59], [60], [61], [62], [63], [64], [65], [66], [67], [68], [69], [70], [71], [72], [73], [74], [75], [76], [77]]. The time points for data collection ranged from day 18 of gestation to 1 year postnatal. Data collection time points varied between studies and are described in Table 2 and visualized in Figure 2. Of interest, 8 studies only studied male offspring [39,50,52,57,63,73,75,76] and 2 studies only studied female offspring [66,74].

**TABLE 1:** Overview of selected article characteristics (*n* = 37 total).

Characteristic	Output
Year	• 1990–1999 (5%, *n* = 2) • 2000–2019 (57%, *n* = 21) • 2020–2024 (38%, *n* = 14)
Geography	• Oceania (22%, *n* = 8) • North America (22%, *n* = 8) • Asia (24%, *n* = 9) • Europe (14%, *n* = 5) • South America (8%, *n* = 3) • Africa (3%, *n* = 1) • Other: Turkey (8%, *n* = 3)
Species	• Sprague–Dawley rats (51%, *n* = 19) • C57BL/6J mice (22%, *n* = 8) • Wistar rats (24%, *n* = 9) • Swiss mice (3%, *n* = 1)
Fructose exposure timing	• Dams pregnancy only (35%, *n* = 13) • Dams pregnancy and lactation (32%, *n* = 12) • Dams pregnancy and lactation, and offspring postweaning (5%, *n* = 2) • Dams prepregnancy and pregnancy (3%, *n* = 1) • Dams prepregnancy, pregnancy, and lactation (16%, *n* = 6) • Dams prepregnancy, pregnancy, and lactation and offspring postweaning (3%, *n* = 1) • Dams pregnancy and offspring postweaning (3%, *n* = 1) • Dams pregnancy and pregnant offspring (3%, *n* = 1)
% Fructose	• 10%–29% wt/vol Solution (48% *n* = 18) • 100 g/L (8%, *n* = 3) • 10%–29% Solution (8%, *n* = 3) • 30%–65% Solution (11%, *n* = 4) • 50%–70% wt:wt (8%, *n* = 3) Other: • 20% of Caloric intake (8%, *n* = 3) • 45%–60% of Carbohydrate intake (5%, *n* = 2) • 34.77 mm (3%, *n* = 1)
% Studies supporting maternal hepatic adiposity (of 14 relevant articles)	• 93% (*n* = 13)
% Studies supporting offspring hepatic adiposity (of 20 relevant articles)	• 84% (*n* = 16)
% Studies supporting maternal whole-body adiposity (of 12 relevant articles)	• 75% (*n* = 9)
% Studies supporting offspring whole-body adiposity (of 11 relevant articles)	• 63% (*n* = 7)

**TABLE 2:** Summary of findings from all included articles (*n* = 37).

Author, year	Country	Species studied	% Fructose solution exposure and timing	Age of data collection	Hepatic adiposity findings	Whole-body adiposity findings
Saad et al., 2016 [72]	United States	C57BL/6J Mice	10% (wt/vol) Fructose solution during pregnancy only	Offspring weighed at birth and 1 y of age	Offspring: female had higher liver fat infiltrates (not significant in males).	Dams: no difference in maternal weight. Offspring: higher percent visceral adipose tissue in female offspring. no difference in weight at birth, but female offspring weights were significantly higher at 1 y of age (both findings were not significant in males).
Jen et al., 1991 [53]	United States	Sprague–Dawley rats	50% Fructose during pregnancy and lactation	Dams killed 2 d after weaning, offspring weight measured at birth	Dams: higher PEPCK enzyme concentrations (rate-limiting enzyme in gluconeogenesis). Livers weighed less than reference group 2 d after weaning.	Dams: no difference in maternal body weight. Fructose-fed dams had higher triglyceride (TG) concentrations on day 10 of gestation. Control dams and sucrose–fed dams had higher TG concentrations during late gestation and weaning, respectively. Offspring: Significantly lower birth weights.
Rawana et al., 1993 [71]	United States	Sprague–Dawley rats	100 g/L Fructose water throughout pregnancy and lactation	Offspring outcomes measured at birth and weaning (day 21). Some dams sacrificed on day 19 of pregnancy, others 5 d after weaning.	Dams: higher liver weight during pregnancy. Higher hepatic lipid concentrations before delivery, but by end of lactation no difference. Higher PEPCK enzyme concentrations on day 19 of pregnancy.	Dams: gained significantly more weight during lactation (no difference during pregnancy) than control. Higher total body fat and body fat relative to body weight during pregnancy compared with control. Higher plasma TG concentration on day 19 of pregnancy. Offspring: pups weighed significantly less than controls at birth and weaning. No difference in total or relative fat contents. Higher plasma TG concentrations.
Ching et al., 2011 [52]	China	Sprague–Dawley rats	60% (wt:wt) Fructose prepregnancy, during pregnancy, lactation, and offspring (only reported data for male offspring)	Offspring weight measured at birth. Other outcomes measured at 14 wk (for pups weaned on control) and 23 wk (for pups weaned on fructose). Dams sacrificed postweaning.	Dams: higher liver weight, hepatic TGs, and cholesterol. Offspring: Male offspring had reduced liver antioxidant enzymes. Liver weights did not differ. Greater hepatic TG in control (dam)/fructose (pup) group and F/C group. Male offspring in fructose (dam) /fructose (pup) group had elevated FFA concentrations, altered lipid metabolism with decreased expression of PPARalpha and PGC1–alpha in both C (dam)/F (pup) and F(dam)/F (pup) groups. In control (dam)/fructose (pup) group increased expression of SREBP1, ACC2, and FAS.	Dams: no difference in body weight during pregnancy or lactation. Higher serum TGs at the end of lactation. Offspring: No significant effect on offspring weight at birth, 14 or 23 wk, higher TG concentrations at 14 weeks in fructose (dam)/control (pup) group or fructose (dam)/ fructose (pup) groups.
Zou et al., 2012 [42]	United States	Sprague–Dawley rats	63% Fructose throughout pregnancy and lactation	Offspring and dams killed at day 21 postpartum (offspring weight was also measured at birth).	Dams: increased liver weight and TG content at weaning. Histology showed lipid droplet accumulation in the liver. Decreased hepatic PGC1alpha expression and (lower) FAS at weaning. Increased PEPCK-C.	Dams: decreased body weight gain. Offspring: No effect on offspring birth weight, lower weaned weight.
Rodriguez et al., 2013 [70]	Spain	Sprague–Dawley rats	10% (wt/vol) Fructose throughout pregnancy only	Measurements taken on the 21st d of pregnancy	Dams: hepatic steatosis and high hepatic TGs. Enzymes involved in fatty acid synthesis increased (e.g., ChREBP). Higher expression of lipogenic genes like FAS and acetyl-coA carboxylase (ACC). Fetus: low TG compared with control. Fetal liver shows hepatic steatosis, increased SREBP1 and SCD1. Lower UCP2 (less hepatic fatty catabolism) Fatty acid catabolism diminished.	Dams: no weight changes. High serum TGs. Fetus: no effect on weight.
Mukai et al., 2013 [69]	Japan	Wistar Rats	100 g/L fructose throughout pregnancy only	Measurements taken on 20th d of pregnancy	Dams: liver weight significantly higher than control and higher hepatic TG concentrations were found. Histopathology revealed lipid accumulation in hepatocytes around central vein. Increased SREBP-1c mRNA expression, high FAS mRNA expression in maternal but not fetal liver reported. PPARalpha concentrations not statistically significant while acyl-CoA oxidase concentrations were significantly lower. Fetus: no significant difference for fetal hepatic TG concentrations. High SREBP1c mRNA expression seen.	Dams: no difference in body weights from day 0 to day 19. Fetus: no significant difference in fetal weights.
Mukai et al., 2014 [68]	Japan	Wistar Rats	100 g/L fructose throughout pregnancy only	Some dams killed on gestational day 21, pups killed and analyzed on postnatal day 22.	Offspring: no difference in liver weights. AMPK phosphorylation and Sirt1 expression was reduced in liver of female offspring and GP6ase activity was increased (not seen in males).	Dams: no difference in body weight on gestational day 21. Fetus: no difference in fetal weight on gestational day 21. Offspring: no difference in weight (female and male) after birth or postnatal day 22.
Toop et al., 2015 [67]	Australia	Wistar rats	10% (w/v) HFCS-55 prepregnancy, during pregnancy, and lactation	Offspring measurements taken at birth and 21 d postnatal. Unclear when data in tables was taken. Dam measurements taken postweaning.	Dams: no difference in liver weights. Offspring: lower relative liver weight.	Dams: no significant difference in body weight or in visceral and total fat mass. No significant difference in plasma TG concentrations.
Li et al., 2015 [79]	New Zealand	Wistar rats	20% Calories from fructose throughout pregnancy and lactation	Neonatal outcomes measured at birth; maternal outcomes measured at the end of lactation.	Dams: liver weight significantly increased and reversed by taurine supplementation. Macrovesicular and microvesicular steatosis observed. Significantly higher NAS (nonalcoholic fatty liver disease activity) score. Increased SREBP1c, FASN, and fructokinase hepatic gene expression. Decreased PPARalpha, PEPCK, PGC1alpha. Offspring: Increased hepatic PEPCK expression in female and male neonates.	Dams: significantly increased maternal body weight gain but no difference in total fat. Significant increase in maternal plasma TGs, FFAs and cholesterol.
Sari et al., 2015 [66]	Turkey	Sprague–Dawley Rats	20% Fructose prepregnancy, during pregnancy and lactation. Female offspring only	Dams killed and measurements taken after weaning (21 d). Offspring measurements taken at day 50 postnatal.	Offspring: increased intensity of COX-2 staining in the liver (early phase of inflammation)	Dams: no difference in body weights or in serum TG and cholesterol concentrations. iNOS intensity was increased in retroperitoneal adipose tissue (early phase of inflammation) Offspring: higher mean body weight and increased serum TGs. Increased intensity of iNOS staining in the retroperitoneal adipose tissue and both iNOS and COX-2 staining in the kidney.
Clayton et al., 2015 [40]	Australia	Wistar rats	20% (Total calorie intake) fructose during pregnancy	Offspring and maternal samples collected at embryonic days 21 and 10 postnatal.	Dams: higher liver weights. No significant changes in hepatic TG. Maternal SREBP1c mRNA concentrations were increased at embryo day 21 and PPARalpha elevated at postpartum day 10. Fructose-induced maternal hepatic endoplasmic reticulum stress but does not induce hepatic inflammasome–induced proinflammatory signaling. Fetus: increased hepatic lipid content. Offspring: increased hepatic TG. Females at postnatal day 10 were most vulnerable to prenatal fructose exposure showing a decrease in SIRT1 (upstream of SBREP1c). Higher SREBP1c concentrations in postnatal day 10 in both males and females. In males, genes involved in beta-oxidation of FFA were downregulated (ACAT1, Crot, Acsl4, etc). Thus, in male neonates hepatic FFA oxidation is decreased in favor of lipogenesis.	Dams: no difference in body weight, fat mass or lean mass. Fetus: no effect on body weight or growth. Offspring: no effect on body weight or growth.
Shortliffe et al., 2015 [51]	United States	Sprague–Dawley rats	60% Fructose-enriched feed pellets during pregnancy	Samples were taken 10–13 d into pregnancy (dams only), within 24 h of delivery and a week later.	Dams: hydropic degeneration of livers with microvesicular and macrovesicular fatty infiltration in hepatocytes and leukocyte infiltration. Greater hepatic steatosis, microvesicular fat infiltration, cellular ballooning, and fibrosis than controls.	Dams: fructose-fed dams gained more weight by mid pregnancy, but this lost significance at delivery and postpartum. Higher TG concentrations during pregnancy; normal within a week of delivering. Offspring: No differences in combined offspring weight between groups.
Lineker et al., 2016 [64]	Canada	Wistar rats	10% Fructose solution during pregnancy	Gestational bodyweights of dams measured on GD20, offspring samples taken at 3 (postweaning) or 6 mo of age.		Dams: higher retroperitoneal fat pad and serum TGs. No significant difference in bodyweight. Fetus: no differences in fetal weight or placental weight on GD20. Offspring: male offspring weighed significantly more than female offspring and had higher retroperitoneal fat mass at 3 but not 6 mo of age (not significantly different from control group though).
Alzamendi et al., 2016 [63]	Argentina	Sprague–Dawley rats	10% (wt/vol) Fructose solution during pregnancy. Male offspring only	Offspring samples taken from birth to 80 d postnatal.		Dams: bodyweight throughout pregnancy was similar across both groups. Adult male offspring: no difference in body weight at birth. Retroperitoneal adipose tissue mass not different at 30 d of age but was significantly lower in fructose rats at 60 d of age. However, retroperitoneal adipocyte diameter, area and volume were higher in fructose pads, which suggests adipocyte hypertrophy at 60 d. Lower serum TG concentrations at day 60. Adipocyte area in μm^2^ 1182.24 + 10.64 in control and 2057.79 + 23.57 in fructose group. The finding was significant.
Rodriguez et al., 2016 [74]	Spain	Sprague–Dawley rats	10% wt/vol Fructose solution during pregnancy and in adult female offspring only	Samples taken at days 240 and 261	Offspring: higher liver weight and hepatic TG. SREBP1c, ChREBP and FAS expression increased, but not significantly. PEPCK, G6Pc, and PPARalpha expression was decreased (G6Pc not significant) at day 261.	Offspring: no changes in body weight, higher plasma TGs at day 261. Higher plasma TGs at day 240, but not significant.
Yuruk and Nergis-Unal, 2017 [61]	Turkey	Sprague–Dawley rats	100% Free fructose or HFCS – 55% fructose before mating, during pregnancy and lactation periods. The 3 groups were administered fructose (containing 100% free fructose), high-fructose corn syrup (containing 55% free fructose; HFCS-55), or sucrose (containing 50% bound fructose) added to water for a final concentration of 0.2 g/mL (20%w/v)	Samples taken at the end of lactation period postnatal day 21.	Dams: liver TG highest in group fed 100% fructose, followed by HFCS group. High ACC1 in 100% fructose and HFCS groups indicating more active fatty acid synthesis pathway. Offspring: Highest liver TG in 100% fructose group, followed by HFCS group. High ACC1 in 100% fructose and HFCS groups indicating more active fatty acid synthesis pathway.	Dams: highest body weight in HFCS group, followed by 100% fructose group (HFCS significantly higher than controls). Plasma TG concentrations highest in the 100% fructose group, followed by HFCS (both significantly higher than controls). Offspring: Highest body weight in HFCS group followed by 100% fructose group (HFCS significantly higher than controls). Plasma TG concentrations highest in the 100% fructose group, followed by HFCS (both significantly higher than controls).
Carapeto et al., 2018 [82]	Brazil	C57BL/6J Mice	45% of Carbohydrates from fructose prepregnancy, during pregnancy and lactation. Both mothers and fathers also received experimental diet	Parents were sacrificed after weaning of offspring and offspring were sacrificed at 3 mo of age. Body mass was measured weekly.	Dams: higher liver weight, enlarged liver due to significant increase in hepatic TGs. Offspring: no significant difference in liver mass or hepatic TGs. Hepatic inflammation in the offspring by activation of Nkkbeta, TNFalpha, SOCS3, and JNK. Increased hepatic lipogenesis in offspring due to overexpression of SREBP1c and FAS.	Offspring: no difference in body mass or plasma TG.
Kaur et al., 2018 [60]	Australia	Wistar rats	10% w/v HFCS–55 fructose during pregnancy and lactation	Offspring samples measured at 3 and 12 wk of age.	Offspring: no difference in relative liver weight between groups at 3 or 12 wk independent of sex. Hepatic omega-7 and omega-9 monounsaturated fatty acids were increased at 3 wk. No significant changes in cHREBP expression or hepatic ACC1, ApoB, FAS, or SCD1 mRNA expression.	Dams: no difference in body weight during pregnancy or lactation. Offspring: no difference in body weight at 3 or 12 wk.
Olaniyi and Olatunji, 2019 [59]	Nigeria	Wistar rats	10% w/v Fructose given for 19 d during pregnancy	Samples measured at day 19 of treatment (during pregnancy)	Dams: significantly higher hepatic mass and hepatic TGs. Histology of liver shows focus of periportal lymphocytic inflammation.	Dams: significantly elevated body weight and visceral fat mass. Increased plasma TG and total cholesterol.
Plows et al., 2020 [80]	New Zealand	C57BL/6J Mice	20% Of caloric intake from HFCS during pregnancy only	Samples collected at gestational day 18.5		Dams: no difference in body weight. No significant difference in mean adipocyte area between groups, but the percentage of adipocytes in gonadal adipose tissue with an area >1900 μm^2^ was increased in the fructose group. Fetus: no significant differences in fetal weight. Placental weight significantly greater for females but not males compared with control.
Kisioglu and Nergis-Unal, 2020 [58]	Turkey	Sprague–Dawley rats	100% Free fructose or HFCS at 20% (wt/vol) prepregnancy and throughout pregnancy and lactation	Samples collected at the end of lactation period (23-wk-old dams and 3-wk-old offspring)		Dams: total body fat and body fat to weight ratio was higher than control (highest total body fat in HFCS, followed by 100% fructose; highest ratio in 100% fructose followed by HFCS). Offspring: Higher total body fat and body fat to weight ratio (highest in HFCS, followed by 100% fructose).
Yamazaki et al., 2020 [57]	Japan	Sprague–Dawley rats	20% (wt/vol) Fructose throughout pregnancy and lactation. Male offspring only	Samples taken between postnatal days 21 and 160		Dams: no difference in body weight. Offspring: no difference in offspring weights at postnatal day 21. Lower relative body weights at postnatal day 119. No change in serum TG at any time point.
Koo et al., 2021 [56]	Korea	C57BL/6J Mice	20% (wt/vol) Fructose throughout pregnancy and lactation	Offspring analyses performed at 7 mo of age.	Offspring: at 7 mo, had fatty liver, enlarged liver, high hepatic TG. Increased concentrations of lipogenesis proteins in both sexes (SREBP, FAS, SCD1, ACC) were also seen.	Offspring: Higher body weights and serum TGs at 7 mo of age.
Toop et al., 2017 [62]	Australia	Wistar rats	10% (wt/vol) HFCS-55 solution, throughout pregnancy and lactation	Offspring weighed starting from birth to 12 wk, other samples taken at 3 and 12 wk.	Offspring: female offspring who were exposed to fructose during pregnancy and lactation had higher relative liver weight at 12 wk (results not seen in males). HCFS-55 prenatally had no significant effect on hepatic lipid concentrations.	Offspring: no effects on offspring body weight. Exposure to HFCS-55 during the suckling period alone, but not during the prenatal period, was associated with increased relative omental fat mass both sexes at 3 weeks of age. However, by 12 wk, males who were exposed to fructose both during pregnancy and lactation had lower visceral and retroperitoneal fat mass.
Arentson-Lantz et al., 2016 [50]	United States	Sprague–Dawley rats	63% Fructose throughout pregnancy, lactation and post weaning Male offspring only.	Measurements taken at birth, weaning (21 d) and 18 wk of age.	Offspring: had increased liver TGs and expressed greater transcription of hepatic G6pase, PPARalpha, PCK1 (regulator of gluconeogenesis) at 18 wk of age.	Offspring: maternal fructose consumption stunted growth in offspring resulting in lower weight and BMI at 21 d and 18 wk of age regardless of postweaning diet.
Wang et al., 2022 [39]	China	C57BL/6J Mice	20% (wt/vol) Fructose during pregnancy. Studied male offspring only	Subset of dams killed at 18.5 d of gestation; fetuses analyzed then. Some dams also weighed postpartum (unclear when) but all other data collected on day 18.5. Adult offspring samples taken between 7 and 18 wk of age	Dams: hepatic steatosis shown. Fetus: upregulation of ChREBP and its target genes (Fasn, Acc1, and Acly). Offspring: increased liver weight, higher hepatic lipid droplet accumulation and TGs.	Dams: no differences in bodyweight but there was an increase in plasma TG concentrations. Dams showed lipid droplet enlarged brown adipose tissue, but white adipose tissue was less affected. Fetus: no difference in fetal weight, but significant increase in fetal brown adipose tissue weight at the end of gestation. Increased TG concentrations. Offspring: increased body weight (11–18 wk) and fat mass (11–15 wk).
Campbell et al., 2022 [78]	Australia	C57BL/6J Mice	60% Of carbohydrates as fructose prepregnancy and during pregnancy	Dams metabolically profiled before and during pregnancy (half killed at day 18 of pregnancy and the rest at 30 wk of age). Offspring weighed at birth and other sample taken at 12 wk of age (weaned at 3 wk of age).	Dams: higher hepatic TGs than feed pellets-fed and glucose-fed mice on day 18, higher hepatic TGs than feed pellets-fed at 30 wk of age. Fetal: No significant difference in liver weights. Offspring: No significant differences in liver TG concentrations.	Dams: gained less pregnancy weight than feed pellets-fed mice. More subcutaneous, ovarian and retroperitoneal white adipose tissue than feed pellets-fed mice at 30 wk of age. Lower plasma TG concentrations than feed pellets-fed mice at 30 wk of age. Fetal: no significant differences in fetal body weight. Offspring: no significant changes in birth weight or weaning weight. Female offspring had significantly higher body fat percentage at 12 wk of age compared with feed pellets-fed mice (difference not seen in males). Both males and females had larger subcutaneous white adipose tissue depots as a percentage of body weight. Females also had more gonadal white adipose tissue (not seen in males). Both male and females had larger brown adipose tissue depots. No significant difference in plasma TG concentrations in either sex.
Tobar-Bernal et al., 2021 [49]	Mexico	Sprague–Dawley rats	50% (wt/wt) fructose during pregnancy and lactation	Dams sacrificed 3 d postweaning (postpartum day 24) for sample collection. Offspring sacrificed on postpartum day 90 but measurements taken between postpartum days 24–90	Dams: increased liver weight and hepatic TGs. Offspring: no difference in liver weight, but an increase in liver TGs was found. Relative weights of liver tissue in male offspring from both control and fructose groups were lower than in female.	Dams: increased plasma TGs, increased retroperitoneal and parametrial adipose deposits. No difference in bodyweight. Offspring: higher bodyweights for both male and female offspring. Higher plasma TGs, increased weight of retroperitoneal fat pads and gonadal adipose tissue in both sexes compared with standard diet. However, relative adipose gonadal tissue weight was significantly lower in fructose-fed and control males compared with females.
Fauste et al., 2021 [55]	Spain	Sprague–Dawley rats	10% (wt/vol) Fructose in gestation, and pregnancy later in life	Pregnant offspring of fructose-fed dams sacrificed on day 21 of gestation for sample collection.	Offspring: both FF group (due to both an increase in liver TGs and cholesterol content) and FC group (mainly related to cholesterol concentration) had significantly marked liver steatosis. mRNA concentrations of SREBP1c were higher in FC and FF groups, but not statistically significant. SCD1, FAS, ATP-citrate lyase, and hepatic TG tended to be augmented in FC and FF groups (more evident in FF group). Lower expression of CPT1 in the FF group, indicating a reduction of fatty acid catabolism. HMG-CoA expression significantly higher in the FC group compared with FF. Offspring fetuses: the livers of fetuses from FC and FF dams showed significant lipid accumulation. Significant increase in fetal liver cholesterol from FC and FF groups. Fetal liver gene expression followed the same patterns as in FC and FF dams (with the exception of HMG-CoA, which was the same in all groups), in accordance with liver steatosis, but was not significant.	Fetus: pregnant rats from fructose-fed dams displayed clear lipid accumulation in placenta (FC > FF) mainly due to accumulation of TGs.
Bridge-Comer et al., 2022 [81]	New Zealand	C57BL/6J Mice	34.7 mm fructose in drinking water throughout pregnancy and lactation	Dams sacrificed at 9 wk postpartum for sample collection.		Dams: no difference in body weight. No difference in absolute or relative ovarian or gonadal fat tissue weight. A slight decrease in gonadal adipocyte size distribution (%) at 1–2000, and 2–3000 μm^2^, but increased at 6–7000 μm^2^ adipocyte area in comparison to the control suggesting that adipocyte hypertrophy was induced following fructose consumptions across pregnancy and lactation.
Ando et al., 2022 [73]	Japan	Sprague–Dawley rats	20% high-fructose solution during pregnancy and lactation. Looked at male offspring only	Dams killed during weaning period. Offspring weighed weekly postweaning days 21–60. Samples taken from groups at days 21 and 60.	Offspring: no significant difference in hepatic TG. Hepatic PPARalpha and CPT1a protein expression significantly decreased at postnatal day 60.	Dams: significantly higher body weight changes. Offspring: no significant changes in body weight. Increased serum TG at postnatal day 60.
Rodriguez et al., 2015 [65]	Spain	Sprague–Dawley rats	10% (wt/vol) Solution during pregnancy	Samples measured in adult progeny at 261 d of age.	Offspring: no differences in liver weights. Higher hepatic TG content and diminished hepatic expression of fatty acid catabolism enzymes (L–CPT–I and PPAR gammacoactivator 1-alpha (PGC1α) concentrations) in males. The concentration of mRNA for SREBP1c was significantly reduced in males only, PEPCK, G6pc, LPK were not different. Females had similar hepatic TG values and expression of fatty acid catabolism enzymes to control and glucose-fed groups.	Offpsring: no changes in body weight. No differences in epididymal and lumbar adipose tissue, and brown adipose tissue weight. Plasma TG concentrations lower than control (although not significant)
Magenis et al., 2024 [77]	Brazil	Swiss mice	20%/L Fructose during prepregnancy, copulation, pregnancy, and lactation	Dams killed at weaning (body weights measured prepregnancy, at the end of pregnancy and post pregnancy). Offspring data collected at 60 d of age (body weight measured at 7, 14, 21, 30, and 60 d)	Offspring: increase in hepatic steatosis in both male and female offspring. VPE significantly reduced the area of steatosis in relation to the fructose group.	Dams: no significant changes in bodyweight. Offspring: female offspring of fructose-fed dams had significantly higher body weight compared with control at 7, 14, and 60 d of age. Male offspring of fructose-fed dams had higher body weight at days 7, 14, and 21 of age. No significant difference in body adiposity index for female offspring, however, increased in male offspring. Body adiposity index was reduced when fructose-fed dams exercised in the males but not female offspring. (Body adiposity index was calculated by weighing the epididymal, mesenteric, retroperitoneal, and perirenal fats)
Li et al., 2024 [54]	China	C57BL/6J Mice	70% Fructose. During pregnancy (also 10 kcal% from fat)	Some dams sacrificed on day 17, offspring sacrificed at 8 wk of age.	Dams: high-fructose diet in pregnancy upregulated the expression of hepatic ChREBP mRNA and its target genes PKLR and SCD1 (involved in lipogenesis), contributing to the development of hepatic steatosis. Offspring: maternal high-fructose diet during pregnancy strongly increased serum progesterone concentrations and hepatic ChREBP mRNA in female offspring but not in male offspring. Female offspring had higher lipid synthesis–related protein expression. Increase in liver TGs and FFAs in female offspring of fructose-fed dams. Increase in liver TGs but not FFAs in males. Maternal hepatic ChREBP deficiency inhibited the high fructose-induced ChREBP expression in female offspring but had no effect in male offspring.	Offspring: increase in serum TGs and FFAs in female offspring of fructose-fed dams. No significant changes in male offspring.
Ando et al., 2024 [75]	Japan	Sprague–Dawley rats	20% High fructose during pregnancy and lactation. Male offspring only	Body weight of offspring measured weekly PD21–60. Dams killed during weaning period. Samples taken from groups at days 21 and 60.	Offspring: 13 upregulated genes associated with positive regulation of lipid biosynthesis in small hepatocytes that may persist into adulthood (PD60).	Dams: no difference in body weight. Offspring: No difference in male offspring weights.
Fauste et al., 2024 [76]	Spain	Sprague–Dawley rats	10% Fructose wt/vol during pregnancy and adult male offspring (control, fructose 10% wt/vol., fructose+cholesterol). Male offspring only	Male offspring sacrificed at 111 d of age.	Offspring: elevated FAS protein concentrations in descendants receiving fructose but not in those consuming a Western diet. Strong hepatic steatosis seen in offspring fed a Western diet regardless of mother’s diet, but not in fructose diet.	Offspring: body weight significantly lower in fructose-fed dams than those from control-fed dams

Note: FC, maternal fructose exposure and control diet during pregnancy; FF, maternal fructose exposure and exposure again during pregnancy.

FFA, free fatty acid; HFCS, high-fructose corn syrup; ChREBP, carbohydrate response element–binding protein; FAS, fatty acid synthase; SREBP1c, sterol regulatory element–binding protein 1.

### Whole-body adiposity

#### Maternal outcomes

Although 2 studies recorded a decrease in body weight in fructose-fed dams [42,78] and others reported an increase in body weight [59,61,71,73,79], most (20 of 27 studies) reported no difference in body weight in fructose-fed dams compared with control groups [39,40,49,[51], [52], [53],^57^,^60^,^63^,^64^,[66], [67], [68],^70^,^72^,^75^,^77^,^80^,^81^]. Nine of the 12 studies that discussed whole-body adiposity supported our definition of maternal whole-body adiposity in fructose-fed dams (Table 3) [39,40,49,58,59,64,67,71,[78], [79], [80], [81]]. Specifically, studies reported increases in total body fat mass [58,71], adipocyte hypertrophy [81], retroperitoneal [49,64,78] visceral [59,78], or parametrial hypertrophy [49], or mean gonadal adipose tissue area [80], lipid droplet enlarged brown adipose tissue [39], or ovarian adipose tissue mass [78]. However, 4 studies reported no difference in various body fat measures including fat mass or total fat percentage [40,67], visceral adipose tissue mass [67], mean adipocyte area [80], gonadal or ovarian adipose tissue weight [81] in fructose-fed dams. Of note, it was a common finding for fructose-fed dams to have increased serum [52,64,70] and plasma [39,49,51,53,59,71,79] triglyceride concentrations during pregnancy, without changes in body composition.

**TABLE 3:** Summary of studies reporting maternal whole-body adiposity results (*n* = 12).

Association with whole-body adiposity	Fructose exposure (wt:vol or wt:wt)				
			Other:		
	<30%, Low dose	>30%, High dose	20% (Of total calorie intake)	60% (Of carbohydrates)	34.7 mm Fructose in drinking water
Yes (*n* = 9)	Rawana et al., 1993 [71]; Lineker et al., 2016 [64]; Olaniyi and Olatunji, 2019 [59]; Kisioglu and Nergis-Unal, 2020 [58]; Wang et al., 2022 [39]	Tobar-Bernal et al., 2021 [49]	Plows et al., 2020 [80]	Campbell et al., 2022 [78]	Bridge-Comer et al., 2022 [81]
No (*n* = 3)	Toop et al., 2015 [67]		Clayton et al., 2015 [40]; Li et al., 2015 [79]		

#### Fetal outcomes

None of the 8 studies reporting fetal weight found differences between control and maternal fructose-exposed fetuses [39,40,64,[68], [69], [70],^78^,^80^]. Interestingly, 1 study reported a significant increase in fetal brown adipose tissue weight at the end of gestation, despite no change in overall fetal body weight [39]. Additionally, this study also reported higher fetal ChREBP mRNA expression and its target genes (FASN, ACC1, and ACLY) in fetal brown adipose tissue.[39]

In a transgenerational study, pregnant rats from fructose-fed dams exhibited significant placental lipid accumulation, primarily due to triglyceride buildup, regardless of whether they were fed fructose or control diets during their own pregnancy [55].

#### Offspring outcomes

Similar to maternal outcomes, most studies (*n* = 18) reported no significant differences in offspring weight at various time points for fructose-fed dams (Table 2) [40,42,[50], [51], [52],^57^,^60^,[62], [63], [64], [65],^68^,[72], [73], [74], [75],^78^,^82^].

Seven of 11 studies found significant increases in offspring measures of adiposity [39,49,58,63,72,77,78] (Table 4) [39,49,58,[62], [63], [64], [65],^71^,^72^,^77^,^78^]. These studies found increases in total body fat [58,78] brown adipose tissue (in both sexes) [78], visceral adipose tissue [72,77], fat mass [39], subcutaneous fat tissue [78], retroperitoneal [49,77], and gonadal adipose tissue [49,78].^.^ One study reported reduced retroperitoneal adipose tissue mass but increased adipocyte hypertrophy in male offspring at 60 d postnatal (Table 2) [63]. Three studies identified no significant difference in fat content compared with control groups [64,65,71]. One study found that offspring exposure to high-fructose corn syrup during the suckling period, but not the prenatal period, increased relative visceral adipose tissue mass at 3 wk [62].

**TABLE 4:** Summary of studies reporting offspring whole-body adiposity results (*n* = 11).

Association with whole-body adiposity	Fructose exposure (wt:vol or wt:wt)				
			Other:		
	<30%, Low dose	>30%, High dose	20% (Of total calorie intake)	45% (Of carbohydrates)	60% (Of carbohydrates)
Yes (*n* = 7)	Saad et al., 2016 [72]; Alzamendi et al., 2016 [63]; Kisioglu and Nergis-Unal, 2020 [58]; Wang et al., 2022 [39]; Magenis et al., 2024 [77]	Tobar-Bernal et al., 2021 [49]			Campbell et al., 2022 [78]
No (*n* = 4)	Rawana et al., 1993 [71]; Lineker et al., 2016 [64]; Toop et al., 2017 [62]; Rodriguez et al., 2015 [65]				

Similar to the observations of TG concentrations in maternal circulation, results in offspring varied regarding serum triglyceride concentrations. 8 studies reported higher plasma or serum triglyceride concentrations in the offspring of fructose-fed dams [49,52,54,56,66,71,73,74] compared with the 4 that reported lower concentrations [63] or no effect [57,78,82].

### Hepatic adiposity

#### Maternal outcome

In 10 studies, fructose-fed dams were found to have higher liver weight [40,42,49,52,59,69,71,74,79,82]. Only one study reported no change in liver weights [67] and one reported lower liver weights [53]. In the majority of these reports, low-dose fructose consumption (<30% wt:vol or wt:wt) was used (Table 5) [39,40,42,49,51,52,59,61,[69], [70], [71],^78^,^79^,^82^].

**TABLE 5:** Summary of studies reporting maternal hepatic adiposity results (*n* = 14).

Association with hepatic adiposity	Fructose exposure (wt:vol or wt:wt)				
			Other:		
	<30%, Low dose	>30%, High dose	20% (of Total calorie intake)	45% (of Carbohydrates)	60% (of Carbohydrates)
Yes (*n* = 13)	Rawana et al., 1993 [71]; Rodriguez et al., 2013 [70]; Mukai et al., 2013 [69]; Yuruk and Nergis-Unal, 2017 [61]; Olaniyi and Olatunji, 2019 [59]; Wang et al., 2022 [39]	Ching et al., 2011 [52]; Zou et al., 2012 [42]; Shortliffe et al., 2015 [51]; Tobar-Bernal et al., 2021 [49]	Li et al., 2015 [79]	Carapeto et al., 2018 [82]	Campbell et al., 2022 [78]
No (*n* = 1)			Clayton et al., 2015 [40]		

From 14 studies that investigated hepatic lipid accumulation after prenatal fructose exposure, 13 studies supported the association between maternal fructose consumption and maternal hepatic adiposity (Table 5). Specifically, studies reported higher concentrations of hepatic triglycerides [42,49,52,59,61,69,70,78,82], hepatic lipid concentrations [71], and hepatic steatosis [39,51,54,70,79] in fructose-fed dams. In articles that reported histological findings of the liver periportal lymphocytic inflammation [59] and lipid droplets in periportal hepatocytes [42,51,69] were observed.

Elevated mRNA concentrations of key proteins associated with lipid synthesis provided potential mechanisms associated with increased maternal fructose consumption, leading to maternal hepatic adiposity. This was associated with elevated gene expression of enzymes involved in fatty acid synthesis (*e.g*., ChREBP, FAS, acetyl-coA carboxylase, PKLR, SCD1) [54,69,70,79] as well as SREBP1c [40,69,79]. Additionally, PEPCK gene expression, a rate-limiting enzyme in gluconeogenesis, was elevated in the livers of fructose-fed dams [42,53,71]. Of note, one study reported no significant changes in maternal hepatic triglyceride concentrations, however, both maternal and offspring SREBP1c mRNA concentrations were increased, without increased hepatic lipid accumulation [40]. Hepatic ChREBP deficiency improved fructose-induced hepatic steatosis [54].

#### Fetal outcomes

Six studies reported the effects of prenatal fructose exposure on fetal hepatic outcomes (Table 2) [39,40,55,69,70,78]. Of these studies, 5 did not report fetal liver weight,^26^, [40,55,69,70] one study reported no significant difference in liver weight [78]. Three studies reported fetal hepatic steatosis [40,55,70], whereas one study reported no significant differences in hepatic triglyceride concentrations, although expression of hepatic lipogenic proteins (*e.g*., SREBP1c and SCD1) was elevated [69]. One study reported an upregulation of ChREBP and its target genes (e.g., FASN, ACC1, and ACLY).^26^

#### Offspring outcomes

A decrease [67] or no change [49,52,60,65,68,82] in liver weight was reported in the offspring of fructose-fed mothers in 7 studies. Four studies documented an increased liver weight [39,62,74] or enlarged liver [56] in the offspring. Interestingly, hepatic adipose content varied depending on the age of the offspring, with one study reporting increased adipose content at 3 wk of age but no significant changes at 12 wk [62]. Additional information on the age of data collection and results obtained at these time points can be found in Figure 2 and Table 2.

Sixteen of 20 studies that investigated hepatic fat accumulation in the offspring of fructose-fed dams found hepatic steatosis in the offspring (Table 6) [39,40,49,50,52,[54], [55], [56],^60^,^61^,^62^,^65^,^66^,[72], [73], [74],[76], [77], [78],^82^]. Hepatic adipose accumulation [39,40,49,50,52,55,56,60, 61,65,66,72,74,82] was commonly found among offspring of fructose-fed dams, in association with increased expression of lipogenic proteins such as SREBP1c and FAS [40,55,69,74,76,82]. Five studies reported no differences in offspring hepatic triglyceride concentrations [62,73,76,78,82]. Interestingly, Fauste et al. [55] reported an intergenerational effect where pregnant offspring of fructose-fed dams had significantly marked hepatic steatosis in both fructose diets and control diets during pregnancy. Another study found that male offspring from fructose-fed dams that ingested a Western diet (fructose and cholesterol) provoked hepatic steatosis and hepatomegaly that was not seen in offspring from fructose-fed dams that ingested a fructose-only diet [76].

**TABLE 6:** Summary of studies reporting offspring hepatic adiposity results (*n* = 20).

Association with hepatic adiposity	Fructose exposure (wt:vol or wt:wt)				
			Other:		
	<30%, Low dose	>30%, High dose	20% (of Caloric intake)	45% (of Carbohydrates)	60% (of Carbohydrates)
Yes (*n* = 16)	Saad et al., 2016 [72]; Rodriguez et al., 2016 [74]; Yuruk and Nergis-Unal, 2017 [61]; Koo et al., 2020 [56]; Wang et al., 2022 [39]; Fauste et al., 2021 [55]; Rodriguez et al., 2015 [65]; Sari et al., 2015 [66]; Kaur et al., 2018 [60]; Magenis et al., 2024 [77]	Ching et al., 2011 [52]; Arentson-Lantz et al., 2016 [50]; Tobar-Bernal et al., 2021 [49]; Li et al., 2024 [54]	Clayton et al., 2015 [40]	Carapeto et al., 2018 [82]	
No (*n* = 4)	Toop et al., 2017 [62]; Ando et al., 2022 [73]; Fauste 2024 [76]				Campbell et al., 2022 [78]

#### Sex differences

Importantly, several studies reported sex differences in hepatic adipose content, liver weight, and offspring weight. Two studies found that female offspring of dams exposed to 10% wt/vol fructose during pregnancy had higher liver fat infiltrates, greater offspring weight at 1 y, and increased relative liver weights compared with male offspring [62,72]. Similarly, one study found a significant reduction in male, but not female fetal body weight with an associated increase in placental weight in females [80]. Female offspring of fructose-fed mice had significantly higher body fat percentage at 12 wk of age compared with feed pellets-fed mice and had more visceral adipose tissue at 1 y of age when compared with their male counterparts [72,78]. In contrast, one study found that male offspring weighed significantly more than female offspring and had higher retroperitoneal fat mass at 3 mo of age [64]. The higher liver weight found in female offspring of fructose-fed dams in the article by Rodriguez et al. [74] was associated with increased mRNA expression of SREBP1c, ChREBP, and FAS. In another study, the hepatic mRNA concentration of SREBP1c in male offspring was significantly reduced [65]. Li et al. [54] found striking sex differences in the offspring of fructose-fed dams. Female offspring had higher serum triglyceride concentrations and free fatty acids that were not seen in male offspring. Maternal fructose consumption strongly increased serum progesterone concentrations, hepatic ChREBP mRNA expression, and lipid synthesis–related protein expression in female but not male offspring. Furthermore, maternal hepatic ChREBP deficiency ameliorated fructose-induced lipid accumulation and insulin resistance in female offspring but had no effect on male offspring.

Sex differences have also been observed in interventional studies, aimed at mitigating the effects of excessive fructose feeding. Li et al. [79] found that maternal taurine supplementation attenuated maternal fructose-induced hepatic tumor necrosis factor receptor 1 in male offspring but not in female offspring. Taurine supplementation also did not appear to affect the elevated hepatic PEPCK mRNA expression in female offspring, although it did normalize it in male offspring. Furthermore, the expression of SIRT1, an enzyme that inhibits lipogenesis, was significantly reduced in female offspring but not in males [40,68].

## Discussion

In this scoping review, although no human publications met our inclusion criteria, there were a large number of rodent studies reporting information on maternal fructose consumption and the impacts on the development of maternal, fetal, and offspring intra- and extrahepatic adiposity. In these rodent studies, the relationship between maternal fructose consumption and whole-body adiposity in dams and offspring is not consistent. Although most did not show a difference in body weight, many studies did demonstrate an increase in measures of adiposity such as total fat mass and adipocyte hypertrophy. Moreover, maternal fructose consumption was associated with hepatic steatosis in rodent dams, fetuses, and offspring, which is a recognized hallmark of NAFLD. Several studies investigated both female and male offspring and identified sex-specific findings where female offspring were found to be more susceptible to prenatal fructose exposure than males, with greater hepatic adiposity and whole-body adiposity. Although ad libitum access to fructose-containing diets does not necessarily increase body weight, it consistently increases fat mass in rodents [83]. These observations are consistent with our current findings, as most of our studies found no difference in body weight in dams and offspring, however, several articles reported adipocyte hypertrophy and adipose tissue mass increases, which may be early signs of metabolic syndrome. Key regulatory proteins identified in association with the increased hepatic steatosis included underlying mechanisms associated with lipid synthesis including ChREBP [39,54,70,74], FAS [55,56,69,70,74,82], SREBP1c [56,69,74,82,84], and their target genes. Thus, the upregulation of networks involved in hepatic *de novo* lipogenesis may likely play a role in the progression of NAFLD in the mother and its programming effects in the fetus and offspring.

Another potential mechanism in the adverse effects of fructose may be related to the metabolic effects of uric acid [11,38], a byproduct of fructose metabolism [85]. Although this was not a focus in our scoping review, hyperuricemia is a key component of the development of metabolic syndrome [5,6]. Interestingly, GLUT9 knockout in enterocytes in mice induces hyperuricemia and elevated hepatic triglycerides and free fatty acids [11]. High-fructose feeding in guinea pigs before and during pregnancy resulted in offspring with significantly altered serum free fatty acids, and increased concentrations of uric acid and triglycerides, all of which had developed before weaning [86]. Additionally, high-fructose (60% fructose) consumption in mice has been demonstrated to increase placental fructose transport via GLUT9, and subsequent de novo uric acid production by activating the activities of the enzymes AMP deaminase and xanthine oxidase, which resulted in increased lipids and oxidative stress in the placenta [36,87]. Treatment with allopurinol, a xanthine oxidase inhibitor, attenuated such effects [11,36]. Given that these studies highlight an unfavorable impact of excess fructose on various organ systems in the perinatal period, further research is required to elucidate the relationship between fructose metabolism and uric acid production during the perinatal period and later life adipogenesis.

Several studies have highlighted that fetal sex, and thereby placental sex, is a critical factor in determining fetoplacental metabolism and also understanding the effects of fructose exposure on fetal and offspring development. Sex-specific fetal programming could imply that the placenta functions differently following exposure to fructose, depending on the sex of the fetus, leading to varied outcomes in terms of growth, metabolic programming, and long-term health risks for the offspring [88,89].Several reports highlight that placental function and structure in response to maternal fructose consumption are different based on fetal sex. For example, one study reported that placental weights in female fetuses tend to be significantly greater than in males, possibly due to lipid accumulation in the placental tissues [80]. However, contrasting findings have been observed in other reports. For example, one study demonstrated that high maternal fructose intake (20% of the diet as fructose) in rats was associated with decreased placental weights in female fetuses compared with their male counterparts, indicating a potential vulnerability in females to high-fructose diets during gestation [90]. These discrepancies in placental weight changes suggest that the mechanisms underlying placental adaptation to maternal diet might be more complex and influenced by factors beyond just fetal sex, such as dosing and duration of exposure.

Sex-specific responses to maternal fructose exposure are not limited to placental changes but extend into postnatal development and metabolic health. Female offspring exposed to high-fructose diets in utero tend to exhibit increased adiposity, obesity, and hepatic lipid deposition later in life. This finding aligns with the concept of developmental programming where early life exposures, such as maternal nutrition, can predispose offspring to long-term health issues such as metabolic syndrome. In contrast, male offspring exposed to the same fructose-rich maternal environment tend to develop different health complications, such as hypertension and insulin resistance [72]. These divergent sex-specific outcomes underscore the role of sex in determining how prenatal exposures shape health trajectories. The involvement of sex hormones in these processes has been hypothesized, as estrogen and testosterone have known effects on lipid metabolism, insulin sensitivity, and blood pressure regulation [[91], [92], [93]]. In a recent study, it is speculated that high fructose intake overactivates ChREBP, which leads to hyperactivation of the progesterone–PPARy–ChREBP–MTTP–progesterone loop. This activation results in excess progesterone entering the offspring via the placenta, activating the fetal progesterone–ChREBP axis, thus resulting in hepatic steatosis in the female offspring [54]. Female offspring are more susceptible to hepatic steatosis due to the relatively higher expression of progesterone receptors compared with males [94]. Nonetheless, future research should focus on elucidating the molecular pathways influenced by sex hormones and their interactions with fructose, and potential impacts on adiposity development across the early life course.

The current review has also highlighted that there is a lack of data concerning the potential transgenerational impacts of excessive maternal fructose consumption. Transgenerational studies are important to elucidate the generational programming of one adverse pregnancy environment (i.e., excess fructose) on the future metabolic health of their offspring and beyond. Rodriguez et al. [91] found that low maternal fructose intake (10% wt/vol) in pregnant rats provoked long-term impacts such as impaired insulin signal transduction, hyperinsulinemia, and hypoadiponectinemia in adult male, but not female progeny. A further transgenerational study by Fauste et al. [55] found that fructose intake during pregnancy contributes to fetal programming, such that their progeny in future pregnancies develop the same programmed phenotype regardless of fructose exposure. These reports demonstrate that fructose exposure prepregnancy and during pregnancy may have lasting deleterious effects on the metabolic health on future generations, and additional studies are needed to fully understand the potential generational impacts.

Although this scoping review identified potential negative effects of maternal fructose consumption on intra- and extrahepatic adiposity in the mother, fetus, and offspring, it should be noted that there was heterogeneity between study methods and outcomes, which limited generalizability to the human setting. A number of factors contributed to this heterogeneity. Firstly, there is great variability with respect to the dose of fructose consumed, as well as the timing (limiting dosage to specific periods of embryonic/fetal or postnatal development), to which fructose was administered. For example, fructose 10% wt/vol dose consumed by rodents throughout pregnancy and postnatally, mimics how fructose is predominantly ingested in human populations, i.e., fructose-sweetened soft drinks, whereas high concentrations of fructose (30%–60% wt/vol) could be deemed supraphysiological, and do not reflect the average human intake [46,47,95]. Second, although some studies reported on liver weight, they did not record measures of hepatic accumulation, and as such were not included in the analysis of studies that supported hepatic adiposity [53,67]. Similarly, many studies reported body weight but failed to report other measures of whole-body adiposity that might be associated with weight. Finally, in addition to factors contributing to heterogeneity, caution should be taken when interpreting findings from rodent studies to understand the potential of similar impacts in humans. A careful understanding of organ developmental phases between species and the impacts of the timing of fructose exposure is paramount. Furthermore, fructose is rarely consumed in isolation in human populations, and longitudinal Western diet studies may be a more appropriate study to understand the relationship between dietary carbohydrates and the development of hepatic and whole-body adiposity. Together, addressing these limitations in future studies will help us better understand the development of offspring hepatic and whole-body adiposity as it relates to maternal fructose consumption in humans.

In conclusion, this scoping review has demonstrated that in rodent models, fructose consumption before and during pregnancy is associated with maternal, fetal, and offspring whole-body and hepatic adiposity, which may be early signs of metabolic syndrome. Notably, there were no human-based literature studies regarding fructose consumption in pregnancy that addressed this research question. There were differential impacts of maternal fructose consumption on male and female offspring and potential transgenerational effects in rodent models, which are avenues for future research. In conclusion, future studies should address the heterogeneity issue of experimental studies and consider the Western diet as an intervention that is more representative of the human setting. Identifying critical windows of susceptibility and sex-specific molecular pathways involved in the vertical transmission of these effects can inform lifestyle modification recommendations and public health guidelines ultimately reducing the risk of chronic diseases such as NAFLD, obesity, diabetes, and cardiovascular disorders in the offspring and future generations.

## Authors contributions

The authors’ responsibilities were as follows – GZ, TRHR: conceptualized and designed the study; MS: performed the initial and updated search strategy; GZ, TRHR, SC, CG, SG: conducted the screening process; GZ, SC: performed data collection; TRHR: verified data collection; GZ, SC: summarized the data and drafted the first version of the manuscript; and all authors: contributed critically to the interpretation of the data, manuscript revisions, and approved the final manuscript and had full access to the data in the study and take responsibility for the integrity of the data and the accuracy of the analysis.

## Funding

The authors reported no funding received for this study.

## Conflict of interest

None of the authors have any to disclose.
