# Supplementary material for: Fructose Consumption in Pregnancy and Associations with Maternal and Offspring Hepatic and Whole-Body Adiposity in Rodents: A Scoping Review
Source: Curr Dev Nutr. 2024 Nov 27;9(1):104510. doi: 10.1016/j.cdnut.2024.104510 (PMC11782591; doi:10.1016/j.cdnut.2024.104510)
Supplement: Multimedia component 1 [file mmc1.docx]

**Fructose consumption in pregnancy and associations with maternal and offspring hepatic and whole-body adiposity in rodents: a scoping review**

Grace Zhao

**APPENDIX A: Search Strategy**

**Medline (OVID)**

Search conducted on November 17^th^, 2021:

1463 TOTAL results

Updated search conducted on August 23^rd^, 2022:

113 results from search/58 duplicates removed by Covidence/ 55 TOTAL new Medline results added.

Updated search conducted again on October 4^th^, 2024:

158 results from search/129 duplicates removed by Covidence/29 TOTAL new Medline results

*search was limited to 2022-current.

**Embase (OVID)**

Search conducted on November 22nd, 2021:

1679 results from search/924 duplicates removed by Covidence/755 TOTAL Embase results.

Updated search conducted on August 23^rd^, 2022:

92 results from search/79 duplicates removed by Covidence/ 13 TOTAL new Embase results added.

Updated search conducted again on October 4^th^, 2024:

208 results from search/37 duplicates removed by Covidence/ 171 TOTAL new Embase results

*search was limited to 2022-current.

**Cochrane Library**

Search conducted on November 22^nd^, 2021:

84 results from search/ 36 duplicates removed by Covidence/ 48 TOTAL results from Cochrane.

Updated search conducted on October 4^th^, 2024:

7 results from search/3 duplicates removed by Covidence/4 TOTAL new results from Cochrane

**Ovid MEDLINE**

1 fructose.tw. or High Fructose Corn Syrup/ or Fructose/

2 "High Fructose Corn Syrup".tw.

3 pregnancy/ or maternal-fetal exchange/ or placentation/

4 (pregnancy or "maternal-fetus exchange" or placenta*).tw.

5 Placenta/

6 Fetus/ or fetus.tw.

7 neonat*.tw. or Infant, Newborn/

8 offspring.tw.

9 Maternal Exposure/ or maternal.tw.

10 fetal.tw. or Fetal Development/

11 newborn.tw.

12 1 or 2

13 3 or 4 or 5 or 6 or 7 or 8 or 9 or 10 or 11

14 12 and 13
